# Supplementary material for: Vitamin D Receptor Gene Polymorphism Predicts the Outcome of Multidisciplinary Rehabilitation in Multiple Sclerosis Patients
Source: Int J Mol Sci. 2023 Aug 29;24(17):13379. doi: 10.3390/ijms241713379 (PMC10487750; doi:10.3390/ijms241713379)
Supplement: Supplementary file 1 [file ijms-24-13379-s001.zip › ijms-2536711-supplementary.pdf]

		EDSS T0 median	IQR	EDSS T1 median	IQR	repeated measure pvalue	mBI T0 median	IQR	mBI T1 median	IQR	repeated measure p value	NRS T0 median	IQR	NRS T1 median	IQR	repeated measure p value
pMS	rs731236 TaqI															
	TT	6,5	1	6,5	0,5		62	26,3	71,5	26,3		5	4	3	4	
	TC	7	1,5	7	1,5		51,5	26,8	61,5	29		5	3,3	2	4	
	CC	7	1,5	6,5	1,5		58	38	66	39		6	3	3	5	
						ns					ns					ns
	rs7975232 3 ApaI															
	AA	7	1,5	6,5	1		60	31	66	27		5,5	3	3	4	
	AC	7	1,5	6,5	1,5		56	32	62	33		5	3	2	4	
	CC	6,5	0,5	6,5	0,5		60	29,5	71	26,5		6	4	3	4	
						ns					ns					ns
	rs10735810 FokI															
	CC	6,5	1,5	6,5	1,8		61	32	65	32		5	4	3	4	
	CT	7	1,5	6,5	1,5		54	29	61	35		5	3	3	4	
	TT	6,5	1,5	6,5	1		58	32	68	25,5		5	6	2	4	
RRMS						ns					ns					ns
	rs731236 TaqI															
	TT	6	1	6	1,5		71	12,5	83	12		7	4	**3**	**5**	
	TC	6	1,4	6	1,5		78,5	8	83,5	9,75		4,5	2,75	**3**	**4**	
	CC	6	1,5	5,5	2		76	12	82	13		6	3	3	2	
						ns					p<0,001					ns
	rs7975232 3 ApaI															
	AA	6	1,5	5,5	1,9		76	11,5	82,5	10,5		6	3	3	2,5	
	AC	6	1,5	6	1,5		74,5	15,3	84	12,5		5	4	3	4	
	CC	6	0,9	6	1,4		70	13,5	80,5	7,8		7	3	2,5	28	
						ns					p=0,01					ns
	rs10735810 FokI															
	CC	6	0,5	5	1		72,5	15,8	81,5	8,2		5	3	3	2,5	
	CT	6	1,5	5,5	2		75	13,5	8	12		5	3	2	4	
	TT	6	1	5,5	1,9	ns	79	9,8	87,5	12,5	ns	5,5	3,5	3	4,8	ns

Table S1: EDSS, mBI and pain NRS values at the admittance (T0) and after MDR (T1) were reported as median and Interquartile range (IQR), Pair data analysis for repeated measure was performed in pMS and RRMS patients in relation to VDR SNPs . Significant p values are reported for TaqI and ApaI genotypes in RRMS patients $p < 0.001$ and $p = 0.01$ respectively.
